# Evaluation of Data‐Based Estimates of Anthropogenic Carbon in the Arctic Ocean

**DOI:** 10.1029/2020JC016124

**Published:** 2020-06-07

**Authors:** J. Terhaar, T. Tanhua, T. Stöven, J. C. Orr, L. Bopp

**Affiliations:** ^1^ Laboratoire des Sciences du Climat et de l'Environnement, LSCE/IPSL, CEA‐CNRS‐UVSQ Université Paris‐Saclay Gif‐sur‐Yvette France; ^2^ Biogeochemistry and Earth System Modelling, Department of Geoscience, Environment and Society Université Libre de Bruxelles Bruxelles Belgium; ^3^ GEOMAR Helmholtz Centre for Ocean Research Kiel Kiel Germany; ^4^ Laboratoire de Météorologie Dynamique, LMD/IPSL, Ecole Normale Supérieure / PSL Université, CNRS, Ecole Polytechnique Sorbonne Université Paris France

**Keywords:** Arctic Ocean, Anthropogenic carbon, Ocean acidification, Transient Time Distribution, CFC‐12

## Abstract

The Arctic Ocean is particularly vulnerable to ocean acidification, a process that is mainly driven by the uptake of anthropogenic carbon (C_ant_) from the atmosphere. Although C_ant_ concentrations cannot be measured directly in the ocean, they have been estimated using data‐based methods such as the transient time distribution (TTD) approach, which characterizes the ventilation of water masses with inert transient tracers, such as CFC‐12. Here, we evaluate the TTD approach in the Arctic Ocean using an eddying ocean model as a test bed. When the TTD approach is applied to simulated CFC‐12 in that model, it underestimates the same model's directly simulated C_ant_ concentrations by up to 12%, a bias that stems from its idealized assumption of gas equilibrium between atmosphere and surface water, both for CFC‐12 and anthropogenic CO_2_. Unlike the idealized assumption, the simulated partial pressure of CFC‐12 (*p*CFC‐12) in Arctic surface waters is undersaturated relative to that in the atmosphere in regions and times of deep‐water formation, while the simulated equivalent for C_ant_ is supersaturated. After accounting for the TTD approach's negative bias, the total amount of C_ant_ in the Arctic Ocean in 2005 increases by 8% to 3.3 ± 0.3 Pg C. By combining the adjusted TTD approach with scenarios of future atmospheric CO_2_, it is estimated that all Arctic waters, from surface to depth, would become corrosive to aragonite by the middle of the next century even if atmospheric CO_2_ could be stabilized at 540 ppm.

## Key Points

- The amount of anthropogenic carbon in the Arctic Ocean appears to be underestimated by 7–12%
- A refined approach yields a new estimate of 3.3 ± 0.3 Pg C for year 2005
- All Arctic Ocean waters become corrosive to aragonite if atmospheric CO2 is held at 540 ppm

## 1 Introduction

Ocean uptake of anthropogenic carbon (C_ant_) drives reductions in ocean pH and carbonate ion concentration, the latter of which reduces the calcium carbonate (CaCO_3_) saturation state (Orr et al., 2005). Surface waters in the high latitudes have naturally lower CaCO_3_ saturation states; hence, as atmospheric CO_2_ climbs, they are more susceptible to reaching saturation states below which they become undersaturated with respect to aragonite, a metastable form of CaCO_3_ (Orr et al., 2005; Steinacher et al., 2009). Unlike in almost all other regions, surface waters in the Arctic Ocean are already partly undersaturated with respect to aragonite particularly near river mouths because of lower alkalinity freshwater input (Chierici & Fransson, 2009; Mathis et al., 2015; Semiletov et al., 2016). The Arctic Ocean's annual‐mean conditions are projected to become entirely undersaturated with respect to aragonite by 2080 under the SRES A2 scenario (Steinacher et al., 2009).

The Arctic's aragonite saturation horizon (ASH), above which waters are supersaturated with respect to aragonite and below which they are undersaturated, is located at around 1,900 m (Anderson et al., 2010). The Arctic ASH is projected to shoal by ∼100 m during the 21st century based on a coupled carbon‐climate model forced under the SRES A2 scenario (Steinacher et al., 2009). The same model projects that the aragonite undersaturation in surface waters will propagate downwards rapidly, merging with the shoaling ASH at about 1,800 m by the end of the century. Conversely, a data‐based approach that imposes exponentially increasing atmospheric CO_2_ suggests that the deep ASH will shoal by much more (∼900 m) and will merge with the deepening undersaturation from the surface at about 1,000 m by the end of the century (Anderson et al., 2010).

The main driver of acidification in the open ocean is the increase in atmospheric CO_2_ during the industrial era and the resulting uptake of C_ant_ by the ocean. Although this absorbed C_ant_ cannot be measured directly, it has been estimated from other oceanographic data by various methods, such as the transit‐time distribution (TTD) approach (Khatiwala et al., 2013; Waugh et al., 2006). For instance, the inverse Gaussian‐TTD (IG‐TTD) method, a specific solution of the TTD framework, aims to constrain the invasion of C_ant_ into the interior ocean by using observations of transient tracers such as CFC‐12, the tritium‐helium pair (^3^H/^3^He), and SF_6_ (Hall et al., 2002; Waugh et al., 2004). With surface boundary conditions of the chosen tracer (e.g., CFC‐12) as well as subsurface measurements of the same tracer, temperature (*T*), and salinity (*S*), the mean age of the water parcel can be determined by the IG‐TTD approach. The resulting mean age is then used in combination with the atmospheric CO_2_ history to estimate the C_ant_ concentration in the respective water parcel.

The TTD approach was first applied to the global ocean by Waugh et al. (2006), who estimated a global ocean C_ant_ uptake of 134 Pg C in 1994. Waugh et al. did not apply the TTD approach in the Arctic because tracer observations there were sparse. Later, with the advent of more tracer observations, Tanhua et al. (2009) applied the TTD method to the Arctic Ocean, estimating that it contained 2.5–3.3 Pg of anthropogenic carbon in 2005. From the TTD estimates of C_ant_ concentrations along the 2005 Beringia section, Anderson et al. (2010) estimated that the Arctic Ocean's average depth of the ASH had shoaled by ∼190 m during the industrial era.

The skill of related data‐based approaches has been tested in general ocean circulation models (Matear et al., 2003; Matsumoto & Gruber, 2005). In a similar fashion, the TTD approach can be applied to simulated CFC‐12 concentrations to obtain a TTD‐based estimate of C_ant_ in the same model as where C_ant_ is simulated directly (reference C_ant_), for example, using the perturbation approach for C_ant_ from Sarmiento et al. (1992). The first such TTD evaluation by Waugh et al. (2006) indicated that the TTD‐derived C_ant_ is similar to the directly simulated C_ant_ reference in all modeled ocean regions except for the Southern Ocean. The issue in the Southern Ocean stems from its upwelled deeper waters, which are impoverished in CFC‐12 and C_ant_ and spend only about 4 months at the surface before being subducted into the ocean interior (Weiss et al., 1979). Although that period is sufficient for air‐sea equilibration of CFC‐12, such is not the case for C_ant_ whose complete air‐sea equilibration takes longer (Broecker & Peng, 1974). Conversely, the TTD approach assumes equilibrium between the atmosphere and surface ocean for both C_ant_ and CFC‐12. Hence, that data‐based approach systematically overestimates C_ant_ in the Southern Ocean (Matear et al., 2003; Waugh et al., 2006).

In the Arctic Ocean, differences between C_ant_ and CFC‐12 in terms of surface‐water saturation relative to the atmosphere are also thought to cause biases in the TTD‐based C_ant_ estimates. But in that region, the sign and magnitude of these biases have not been evaluated (Tanhua et al., 2009). Recently, Rajasakaren et al. (2019) revised upward the C_ant_ inventory estimate for the Arctic Ocean to 3.6 Pg C in 2005 after accounting for the undersaturation of surface‐water CFC‐12 relative to the atmosphere. However, the associated uncertainty is large. Moreover, they did not account for the related air‐sea disequilibrium for C_ant_, which could be larger in magnitude owing to its longer air‐sea equilibration time. They also changed other parameters of the TTD approach, making it impossible to isolate the effect of differences in surface saturation of CFC‐12. In this study, our goals are (1) to evaluate the TTD method in the Arctic Ocean with an ocean model and (2) to use results from that assessment to adjust the existing TTD data‐based estimate of the Arctic C_ant_ inventory (Tanhua et al., 2009).

## 2 Methods

### 2.1 Arctic Ocean and Its Water Masses

The boundaries of the Arctic Ocean are defined following Bates and Mathis (2009), that is, at the Fram Strait, the Barents Sea Opening, the Bering Strait, and the Canadian Arctic Archipelago (between Smith Sound and Baffin Bay).

Arctic Ocean water masses are also defined based on previous studies (Table S1). The Pacific Water (PW) is divided into (1) the Summer Pacific Water (SPW), having temperatures between −1.0°C and 0.4°C and salinities between 31 and 33 (Bourgain et al., 2013; Steele et al., 2004; Woodgate et al., 2010), and (2) the Winter Pacific Water (WPW), having temperatures below −1.4°C and salinities above 32.4 (Pickart et al., 2005). Water from the Atlantic Ocean splits into two branches. The first branch enters the Arctic Ocean through the Fram Strait at intermediate depths and is generally warmer than 0°C and saltier than 34 (Woodgate, 2013). Being disconnected from the surface, it tends to conserve these characteristics and is thus named Atlantic Water (AW). The second branch enters the Arctic Ocean through the Barents Sea Opening. Being mainly located at the surface, it cools to less than 0°C and its salinity increases to values above 34.6 owing to brine rejection (Midttun, 1985). Consequently, the density of this water mass increases, which drives its descent into the deep Arctic Ocean basins through the St Anna Trough (Gammelsrød et al., 2009; Schauer et al., 2002; Smedsrud et al., 2013). Water masses from this second Atlantic branch are named Barents Sea Water (BSW).

### 2.2 Ocean Model

In this study, we used the global ocean circulation model Nucleus for European Modeling of the Ocean—version 3.2 (NEMO‐v3.2) (Madec, 2008) combined with a single‐tracer perturbation approach to simulate C_ant_ (Lachkar et al., 2007; Palmiéri et al., 2015; Sarmiento et al., 1992). This approach is computationally less expensive than a full biogeochemical model, which facilitates running the physical model at higher resolution. The atmospheric CO_2_ forcing for years 1765–1869 comes from Meinshausen et al. (2017) while that for years 1870–2012 is from Le Quéré et al. (2015). To calculate the anthropogenic change in surface‐ocean partial pressure of CO_2_ (*p*CO_2_), referred to as δ*p*CO_2_, the perturbation approach exploits the nearly linear relationship between δ*p*CO_2_ and the ratio between the corresponding perturbation in total dissolved inorganic carbon (δ*C*_T_) and δ*p*CO_2_ (Sarmiento et al., 1992)

(1) $$\frac{\delta p{\text{CO}}_{2}}{\delta C_{T}}=z_{0}+z_{1}\delta p{\text{CO}}_{2},$$

where *z*_0_ and *z*_1_ are each quadratic functions of surface temperature (° C):

(2) $$z_{0}=a_{0}+a_{1}T+a_{2}T^{2},$$

(3) $$z_{1}=b_{0}+b_{1}T+b_{2}T^{2}.$$

The fitted parameters *a*_0_, *a*_1_, *a*_2_, *b*_0_, *b*_1_, and *b*_2_ are taken from Terhaar et al. (2019, Table 4) and were calculated with the K_1_ and K_2_ dissociation constants from Lueker et al. (2000), the dissociation constant for boric acid from Dickson (1990), and the total boron‐to‐salinity ratio from Uppström (1974). For perturbations in atmospheric CO_2_ of up to 280 ppm (a doubling of the preindustrial level), the relationship between δ*p*CO_2_/δ*C*_T_ and δ*p*CO_2_ is essentially linear under the assumption of constant total alkalinity *A*_T_ (2300*μmolkg* ^−1^) and salinity (35). Hence, it depends only on surface ocean temperature. Rearranging (1) yields

(4) $$\delta p{\text{CO}}_{2}=\frac{z_{0}\delta C_{T}}{1-z_{1}\delta C_{T}},$$

the equation by which simulated surface temperature and δ*C*_T_ are used to compute surface δ*p*CO_2_, as needed to compute the air‐sea C_ant_ flux:

(5) $$F_{C_{ant}}=\alpha k_{w}(\delta p{\text{CO}}_{2}^{atm}-\delta p{\text{CO}}_{2}^{oce}),$$

where *α* is the solubility of CO_2_ (Weiss, 1974), *k*_*w*_ is the gas transfer velocity (Wanninkhof, 1992), and $\delta p{\text{CO}}_{2}^{atm}$ and $\delta p{\text{CO}}_{2}^{oce}$ are the anthropogenic changes in partial pressure in the atmosphere and the surface ocean, respectively. For brevity, $\delta p{\text{CO}}_{2}^{oce}$ is usually indicated here as δ*p*CO_2_. The experimentally derived gas transfer velocity implicitly accounts for some effects of bubbles on the air‐sea C_ant_ flux, but not the direct effect of bubbles on gas saturation anomalies from bubble dissolution (Hamme et al., 2017). While bubbles enhance the gas saturation of less soluble gases, for example, O_2_ (Atamanchuk et al., 2020) and SF_6_ (Stöven et al., 2015), they have little effect on the saturation of CO_2_ and CFC‐12, which are more soluble (Hamme et al., 2017; Stöven et al., 2015). Once transferred to the ocean, C_ant_ was treated as a passive tracer with no internal sources nor sinks.

Despite these approximations, the simulated Arctic Ocean C_ant_ inventory (total mass in the ocean) from the perturbation approach agrees within 3% with the C_ant_ inventory in 2005 simulated by the more costly full biogeochemical approach (Terhaar et al., 2019). This relatively small difference between the two approaches probably results from most of the C_ant_ in the Arctic Ocean interior being taken up in the Atlantic Ocean or the Barents Sea, where the sea surface alkalinity is close to the perturbation approach's assumed 2,300μmol kg^−1^. Thus, alkalinity and salinity anomalies at the Arctic Ocean surface due to freshwater input from rivers and sea ice melt (Chierici & Fransson, 2009; Mathis et al., 2015; Semiletov et al., 2016) have little impact beyond its shallow surface waters. Because of limited computational resources, we used a coarser resolution version of the model (ORCA05, nominal resolution of 0.5°) from 1765 to 1957 and the higher resolution version (ORCA025, nominal resolution of 0.25°) only for the final 1958–2012 period. Also included in the model is CFC‐12, an inert tracer that is simulated from 1932 onwards following the OCMIP‐2 protocol (Dutay et al., 2002) but with the atmospheric boundary conditions from Bullister (2015).

### 2.3 TTD Calculation

The TTD approach was applied following Hall et al. (2002), Waugh et al. (2004), and Tanhua et al. (2009). The concentration of a tracer *c*(*r*,*t*) was calculated at each point *r* and time *t* (in years) using the equation

(6) $$c(r,t)=\int_{0}^{\infty }c_{0}(t-t^{\prime })G_{r}(t^{\prime })dt^{\prime },$$

where *G*_*r*_(*t* *′*) is the commonly applied inverse Gaussian of the Green's function (e.g., Olsen et al., 2010; Tanhua et al., 2009; Waugh et al., 2004; Waugh et al., 2006) and *c*_0_(*t*−*t* *′*) is the tracer's surface‐ocean time history with *t* being the year when the tracer concentration in the water cell is calculated and *t* *′* being the transit time of the water mass in years since it left the sea surface. The surface ocean time history of C_ant_ is calculated as the difference between *C*_T_ at time *t*−*t* *′* and preindustrial *C*_T_ (year 1765), where C_T_ is calculated from the simulated temperature in the water cell, fixed salinity (35), and *A*_T_ (2,300μmol kg^−1^), consistent with the perturbation simulation and the atmospheric *p*CO_2_ in the respective year (Le Quéré et al., 2015; Meinshausen et al., 2017), assuming equilibrium between the atmosphere and the surface ocean. To calculate C_T_ from *A*_T_ and *p*CO_2_, we used the MATLAB version of the routine CO2SYS (van Heuven et al., 2011) with K_1_ and K_2_ dissociation constants from Millero et al. (2002), the dissociation constant for boric acid from Dickson (1990), and the total boron‐to‐salinity ratio from Uppström (1974). The solubility of CO_2_ was calculated following Weiss (1974). For these calculations, nutrient concentrations are assumed to be zero. Although phosphoric and silicic acid systems contribute to *A*_T_ and thus should be corrected for when calculating *C*_T_, their effect is assumed to be negligible when calculating C_ant_, a difference between *C*_T_ at time *t*−*t* *′* and preindustrial *C*_T_, both being biased similarly in the same direction.

For every water parcel *r*, *G*_*r*_(*t* *′*) is expressed as

(7) $$G_{r}(t^{\prime })=\sqrt{\frac{\Gamma^{3}}{4\pi \Delta^{2}{t^{\prime }}^{3}}}exp\left(\frac{-\Gamma {\left(t^{\prime }-\Gamma \right)}^{2}}{4\Delta^{2}t^{\prime }}\right),$$

where Δ represents the width of the TTD and Γ the mean age of the water parcel. The mean age was calculated at each point *r*, assuming Δ/Γ=1, from the measured CFC‐12 concentration at time t (*c*(*r*,*t*)) and the surface ocean CFC‐12 history (*c*_0_(*t*−*t* *′*)) calculated from the atmospheric history of the partial pressure of CFC‐12 (*p*CFC‐12) (Bullister, 2015), assuming equilibrium between *p*CFC‐12 in the atmosphere and that in the surface ocean. The solubility of CFC‐12 was calculated following Warner and Weiss (1985). Based on the mean age, *G*_*r*_(*t* *′*) was derived at every point *r*.

In waters where *p*CFC‐12 is supersaturated with respect to the current atmospheric *p*CFC‐12, *G*_*r*_(*t* *′*) cannot be derived; hence, the TTD method cannot be applied. Oceanic *p*CFC‐12 can exceed atmospheric *p*CFC‐12 because atmospheric CFC‐12 levels have declined since 2002. Thus, when comparing C_ant_ calculated by the TTD method to that which is simulated directly, we masked out model grid cells where ocean *p*CFC‐12 is above atmospheric *p*CFC‐12, amounting to 6.5% of the total Arctic Ocean C_ant_ inventory in 2005 based on directly simulated C_ant_ (section 2.4). While the TTD method could not be evaluated in these supersaturated waters, they are included in data‐based C_ant_ estimate from Tanhua et al. (2009) assuming a mean age of zero.

The ratio of Δ to Γ determines the relative importance of advection versus diffusion, with Δ/Γ<1 indicating a larger advective than diffusive share. Here, we used the typical Δ/Γ ratio of 1.0 (Tanhua et al., 2009; Waugh et al., 2006) as the standard case and assume the related ±1*σ* uncertainty to be ±5% following Tanhua et al. (2009, 2008). Furthermore, we made a series of sensitivity tests over a range of Δ/Γ ratios. When that ratio is varied between 0.6 and 1.8, vertical profiles of $C_{\text{ant}}^{\text{TTD}}$ concentrations averaged over the Arctic Ocean still agree within the applied uncertainty of ±5% (Figure S1). In another study, Rajasakaren et al. (2019) compared mean ages derived from SF_6_ with those derived from CFC‐12 while varying the Δ/Γ ratio to identify the value of that ratio where the mean ages derived from both tracers agreed best. From their comparison, they provide a regionally varying best estimate of Δ/Γ for each Arctic Ocean water mass. Their estimates range from 0.6 for Atlantic Water in the Amerasian basin to 1.2 for waters below the Atlantic Water layer. Their results also indicate that the $C_{\text{ant}}^{TTD}$ concentration uncertainty related to the Δ/Γ ratio is at most ±5% (Rajasakaren et al., 2019, figures  8 and 11) in accord with our uncertainty estimation.

The TTD approach assumes that ocean circulation is in steady‐state, that it can be represented by a 1‐D advection‐diffusion equation (*G*_*r*_(*t* *′*)), and that there is one surface‐ocean source region for both tracers, C_ant_ and CFC‐12. It also assumes that there is conservative mixing between water masses from different source regions and that the air‐sea disequilibrium for both tracers is constant in time. In the standard case for TTD, it is assumed that there is no air‐sea disequilibrium for either tracer.

### 2.4 Evaluation of TTD

To evaluate the TTD method, we applied it to simulated CFC‐12 and temperature from the summer of 2005, that is, the year and months used for the TTD estimate from Tanhua et al. (2009). This TTD‐based C_ant_ is denoted as $C_{\text{ant}}^{\text{TTD}}$. For consistency with the perturbation simulations, the *A*_T_ was fixed at 2,300 μmol kg^−1^, the salinity at 35, and the atmospheric boundary conditions for CO_2_ were identical. The TTD method was evaluated separately for SPW, WPW, AW, and BSW.

The TTD approach could not be evaluated in some parts of the Arctic Ocean because of our simulation strategy, which initialized all variables in the high‐resolution configuration ORCA025 in 1958 with results from the coarse‐resolution configuration ORCA05. Around 41% of the oceanic invasion of the directly simulated anthropogenic carbon ( $C_{\text{ant}}^{NEMO}$) occurs before 1958 (with ORCA05), while only 2% of the oceanic CFC‐12 invasion occurs before 1958 (with ORCA025) because of its more recent perturbation. While this 1958 initialization of ORCA025 with output of ORCA05 poses no problem for the evaluation of the TTD method in relatively young water masses, it biases the evaluation for older water masses where the circulation in ORCA05 differs largely from ORCA025. The largest differences between the two configurations is in the deep Arctic Ocean, where separate CFC‐12 simulations indicate that waters are ventilated to 1,300‐min ORCA05 and to 1,800‐min ORCA025 (Terhaar et al., 2019). Thus, in the combined CFC‐12 simulation (ORCA05 for 1932–1957, then ORCA025 for 1958–2012), simulated CFC‐12 in year 2005 is relatively more abundant below 1,300 m, a signature of ORCA025, when compared to $C_{\text{ant}}^{NEMO}$ (ORCA05 for 1765–1957, then ORCA025 for 1958–2012) in the same year because the anthropogenic carbon invasion began much earlier. That is, $C_{\text{ant}}^{NEMO}$ depends more on the characteristics of the simulated circulation in ORCA05. Hence, in waters below 1,300 m, which represent 54% of the Arctic Ocean volume and 10% of the simulated Arctic Ocean C_ant_ inventory, it is inappropriate to evaluate the TTD method.

Furthermore, the evaluation should only be performed in regions where the model represents the Arctic Ocean circulation reasonably well. That is the case for most parts of the Arctic Ocean above 1,300 m except for the western end of the Canada basin, which receives no AW in the model (Terhaar et al., 2019). Consequently, the simulated CFC‐12 concentrations remain close to zero and well below those observed (Tanhua et al., 2009). Hence, the TTD method is also not evaluated in that region, which represents 6% of Arctic Ocean volume and 10% of the simulated Arctic Ocean C_ant_ inventory. After excluding surface waters that were found to be supersaturated with respect to CFC‐12 (3% of the Arctic Ocean's volume and 7% of its C_ant_ inventory), the TTD evaluation was performed in water masses representing 37% of the Arctic Ocean volume and 69% of the simulated Arctic Ocean C_ant_ inventory. Yet despite this partial coverage, results from the TTD evaluation were expanded upon by classifying each water parcel as a member of one of the Arctic's four dominant water masses and then applying the corresponding corrective factors.

### 2.5 Correction of C_ant_

Each data‐based C_ant_ concentration from Tanhua et al. (2009) was adjusted based on its assigned water mass, determined from its associated physical characteristics (Table S1), and the pertinent water‐mass specific corrective factor from our evaluation of the TTD approach (section 3). Water masses between 250 and 800 m across the Arctic Ocean were mainly identified as AW (Smethie et al., 2000), while those below 800 m were largely identified as WPW (Aagaard, 1981; Swift et al., 1997) or BSW (Jones, 2001; Jones et al., 1995; Smedsrud et al., 2013).

### 2.6 Future C_ant_

Coarse‐resolution ocean models tend to underestimate the storage of C_ant_ in the Arctic Ocean and the resulting shoaling of the deep ASH (Terhaar et al., 2019). Yet 21st century projections with high‐resolution ocean‐biogeochemical models remain prohibitive due to their computational costs. A simpler alternative is to estimate future C_ant_ concentrations based on the TTD approach.

Following Anderson et al. (2010), the TTD approach was used here to estimate C_ant_ concentrations along the Beringia 2005 section over the 21st century, but this time with atmospheric CO_2_ from the four Representative Concentration Pathways RCP2.6, RCP4.5, RCP6.0, and RCP8.5 (Meinshausen et al., 2011) instead of with an exponentially increasing atmospheric CO_2_. The TTD was estimated based on observed CFC‐12, temperature, and salinity, while *A*_T_ was computed from salinity based on an Arctic‐specific relationship (MacGilchrist et al., 2014). The computed TTD from observed CFC‐12 in 2005 (equation 7) was then applied to atmospheric CO_2_ levels for 13 different years spanning from 2014 to 2107 in each of the four RCP scenarios. The resulting ocean C_ant_ concentrations were then adjusted based on the corrective factors that were obtained from the TTD evaluation (section 3).

This TTD‐based approach implicitly assumes no physical changes, for example, in ocean circulation, sea‐ice extent, river discharge, temperature, salinity, and mixed‐layer depth. It also assumes that the air‐sea disequilibrium does not change with time. Furthermore, the TTD approach is based on tracers that started to invade the ocean more recently than anthropogenic carbon. To test if the accuracy of the TTD method changes when being applied to a tracer with a longer atmospheric history, we replicated our TTD‐based future estimates and compared them to model estimates using the computationally efficient, coarse‐resolution version of the model (ORCA2, nominal horizontal resolution of 2°). In ORCA2, we simulated CFC‐12 over 1932–2005 and C_ant_ over 1765–2100. The simulated CFC‐12 concentrations in 2005 were used to derive the TTD. This TTD was then combined with atmospheric CO_2_ levels over 1765–2005 and over 1765–2100 to estimate C_ant_ concentrations in the ocean. Despite the considerably longer atmospheric history of C_ant_ in 2100, the relative uncertainty of the TTD estimated C_ant_ concentrations remains almost the same (Figure S2). Although ORCA2 does not simulate the Arctic Ocean circulation as well as ORCA025 (Terhaar et al., 2019), the relative uncertainty of its $C_{\text{ant}}^{\text{TTD}}$ concentrations in 2005 remains similar to that in 2100 over the range of observed CFC‐12 concentrations and thus over the range of observed mean ages. Thus, it appears that the TTD computed from present‐day CFC‐12 may be used to estimate C_ant_ uptake over 1765–2100 for the idealized case where atmospheric CO_2_ increases but climate and the air‐sea disequilibrium do not change.

### 2.7 Ω_A_ and Undersaturation Index

The aragonite saturation state Ω_A_ was calculated using mocsy (Orr & Epitalon, 2015) with dissociation constants recommended for best practices (Dickson et al., 2007). Combined standard uncertainties in Ω_A_ were computed from the standard uncertainties in the dissociation constants and the solubility product for aragonite (Mucci, 1983) using mocsy's uncertainty propagation routine from Orr et al. (2018). Due to the idealized nature of our simulated future estimates, we neglected measurement uncertainties for the CO_2_ system input variables (*A*_T_ and *C*_T_).

Present‐day Ω_A_ was calculated from observed temperature, salinity, C_T_, *A*_*T*_, total dissolved inorganic phosphorus (*P*_T_), total dissolved silicon (*Si*_T_), and depth along the Beringia 2005 section. Preindustrial Ω_A_ was computed after subtracting the adjusted C_ant_ concentration estimates in 2005 from measured *C*_T_ and assuming that the other variables did not change over the industrial period. For estimates of Ω_A_ over the 21st century, the changes in the C_ant_ concentrations since 2005 were added to *C*_T_ measurements taken in 2005, while also neglecting changes in all other variables.

The basin‐wide undersaturation with respect to aragonite was characterized by the undersaturation index (UI), the fraction of the total Arctic Ocean volume where Ω_A_<1. Here, UI is based on Ω_A_ at the measurement points along the Beringia section, weighted by depth and distance. It is not extrapolated beyond the Beringia section to other parts of the Arctic basin.

## 3 Evaluation of TTD Method in the Arctic Ocean

In the Arctic Ocean, the ORCA025 configuration of NEMO‐PISCES, which is initialized in 1958 with output from the ORCA05 configuration, simulates a total $C_{\text{ant}}^{NEMO}$ inventory of 2.5 Pg C in 2005. That simulated inventory lies at the lower end of the uncertainty range of the data‐based estimate from Tanhua et al. (2009) (2.5–3.3 Pg C in 2005) because of (1) the model's inadequate horizontal extension of Atlantic Waters at intermediate depths and (2) its insufficient deep‐water formation (Terhaar et al., 2019). After masking out cells where the *p*CFC‐12 level in the ocean exceeds that in the atmosphere, owing to the decline in atmospheric CFC‐12 concentrations since 2002, the $C_{\text{ant}}^{NEMO}$ inventory is reduced to 2.3 Pg C. In comparison, the $C_{\text{ant}}^{TTD}$ inventory (calculated from simulated CFC‐12, T, and S) in the Arctic Ocean is 2.2 Pg C.

The difference between $C_{\text{ant}}^{TTD}$ and $C_{\text{ant}}^{NEMO}$ would be expected to be larger had we used ORCA025 before 1958 instead of the coarser configuration ORCA05, which simulates less storage of C_ant_ (Terhaar et al., 2019). Indeed, when evaluating the TTD approach in the model's younger waters that are affected little by the change in resolution in 1958, the calculated $C_{\text{ant}}^{TTD}$ concentrations are seen to underestimate the reference $C_{\text{ant}}^{NEMO}$ concentrations by 7±2% (2.5±0.9μmol kg^−1^ ) in SPW, by 12±3% (4.9±1.3μmol kg^−1^) in WPW, by 4±2% (1.1±0.6μmol kg^−1^) in AW, and by 12±2% (5.0±1.1μmol kg^−1^) in BSW (Table S1).

**Figure 1:**
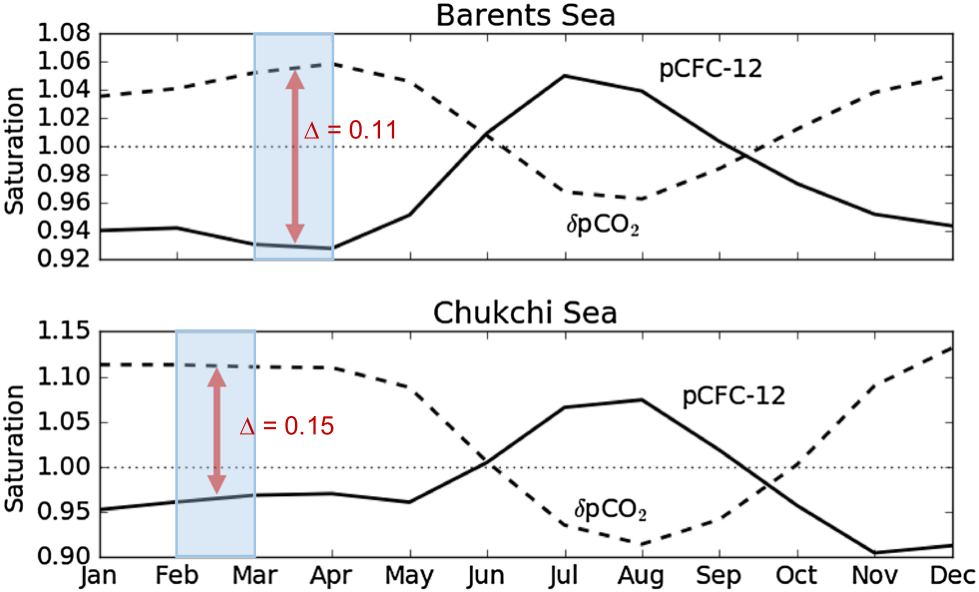
Gas saturation of simulated surface‐ocean δ*p*CO_2_ and *p*CFC‐12 relative to atmospheric values during the seasonal cycle in 2005 shown as averages for the Barents Sea and the Chukchi Sea (regions indicated in Figure 2). The dotted horizontal line indicates air‐sea equilibrium. The shaded blue area indicates the period of deep‐water formation, defined as the period with the lowest density gradient between surface ocean and the deep waters in the Barents Sea and Chukchi Sea. The red arrow indicates the difference in saturation between δ*p*CO_2_ and simulated *p*CFC‐12.

The general underestimation of C_ant_ by the TTD method can be explained by its simplified assumption for the saturation of surface ocean δ*p*CO_2_ and *p*CFC‐12 relative to the atmosphere during deep‐water formation in the Arctic Ocean (Figure 1). Contrary to the TTD assumption of perfect equilibrium between the atmosphere and the surface ocean for both δ*p*CO_2_ and *p*CFC‐12, during deep‐water formation in February and March, simulated δ*p*CO_2_ in the surface ocean is higher than atmospheric δ*p*CO_2_; it is supersaturated. Conversely, surface ocean *p*CFC‐12 is lower than atmospheric *p*CFC‐12; it is undersaturated. This disequilibrium between the surface ocean and atmosphere for both δ*p*CO_2_ and *p*CFC‐12 can be attributed to the rapid heat loss of inflowing PW and AW (Kaltin & Anderson, 2005; Midttun, 1985). For any gas (e.g., CFC‐12), when surface cooling is faster than its air‐sea equilibration, then the dissolved gas concentration changes little and its partial pressure must decline (e.g., see Orr et al., 2017, eq. 21). Conversely, δ*p*CO_2_ (the anthropogenic change in *p*CO_2_) depends on ocean chemistry. Cooling causes oceanic δ*p*CO_2_ to increase because it reduces the carbonate ion concentration and thus the buffer capacity, making the water less able to retain anthropogenic carbon.

Air‐sea equilibration also depends on how long waters remain at the surface and the extent to which those surface waters are ice free. Surface‐water transit times in the Barents and Chukchi Seas are around 6 months each (Loeng, 1991; Spall, 2007). During that transit, the surface waters undergo continual cooling in the Barents Sea and are isolated by substantial sea‐ice cover in the Chukchi Sea. Hence, gas equilibrium between air and sea is not reached, neither for δ*p*CO_2_ nor for *p*CFC‐12, before those surface waters are subducted. Thus, the TTD assumption that both δ*p*CO_2_ and *p*CFC‐12 in those surface waters equilibrate entirely with the atmosphere causes the TTD‐based estimates of C_ant_ concentrations to be too low in the deep waters that were formed from surface waters in the Chukchi and Barents Seas.

**Figure 2:**
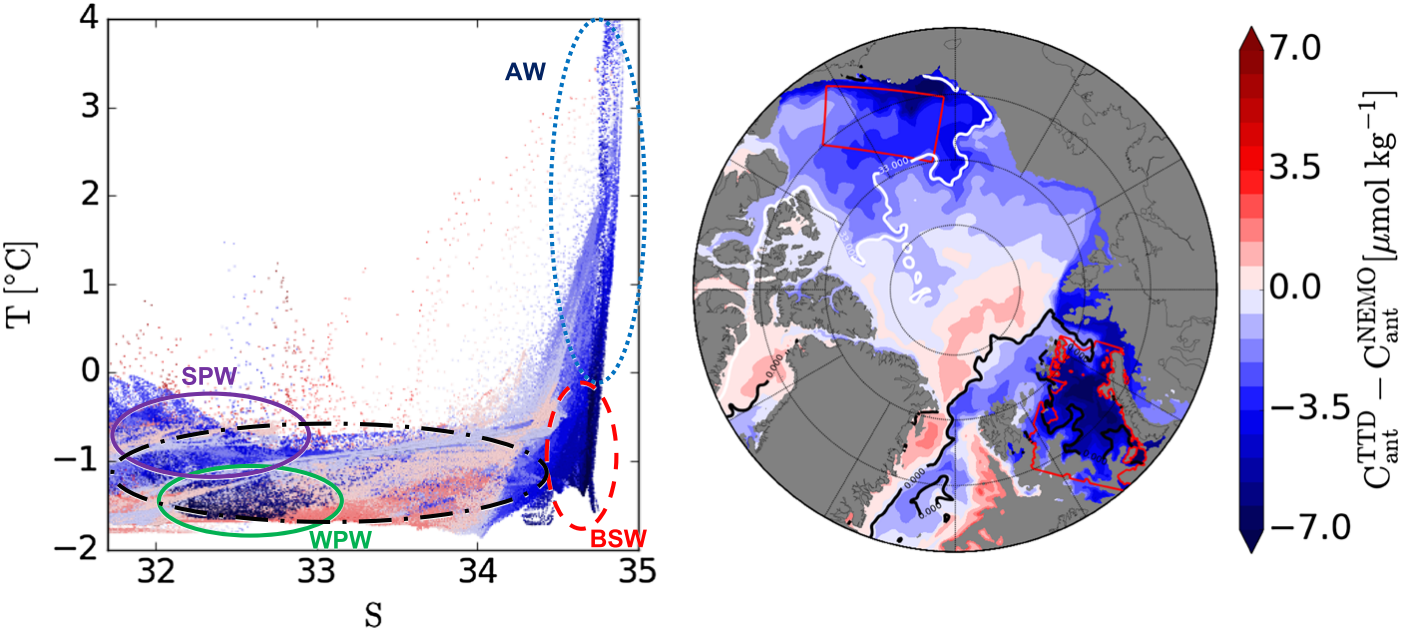
Difference between anthropogenic carbon concentrations $C_{\text{ant}}^{NEMO}$ that are simulated directly ( $C_{\text{ant}}^{NEMO}$) and those that are estimated indirectly by applying the TTD approach in the model ( $C_{\text{ant}}^{TTD}$). Differences are shown for all grid cells in the top 160 m as a function of simulated temperature and salinity (left) and on a map at 112 m, below the stratified surface waters (right). Colored ellipses indicate BSW (red dashed), AW (blue dotted), SPW (magenta solid), WPW (green solid), and the remaining waters close to the surface (black dash‐dotted). The regions in the Chukchi Sea and the Barents Seat that are outlined in red on the map are those for which averages are shown in Figure 1.

The TTD approach's largest underestimates are found in WPW in the Chukchi Sea, delimited by the S = 33 contour in Figure 2, and in the BSW, delimited by the 0°C isotherm in the same figure. The extent of underestimation diminishes as these water masses mix with others (Figure 2). The bias in the BSW is reduced once it enters the Nansen basin and mixes with AW. A signature of this mixing in the model is its decrease in the temperature gradient in the AW along the shelf‐break of the Nansen basin, a feature that is also observed (Gammelsrød et al., 2009; Schauer et al., 2002).

## 4 Correction of Data‐Based Estimate of C_ant_

To refine the data‐based estimate from Tanhua et al. (2009), we adjusted their TTD‐based estimates upward based on the extent of underestimation in each water mass indicated by our model‐based assessment. That is, we assigned corrective factors for the C_ant_ concentrations to each water mass: +4% for AW, +12% for WPW and BSW, and +7% for SPW (section 3, Table S1). After this adjustment of the TTD‐based C_ant_ concentrations, the best estimate for the Arctic Ocean C_ant_ inventory increases from 3.0 to 3.3 Pg C in 2005 while the uncertainty range changes from 2.5–3.3 to 3.0–3.6 Pg C. The lower bound of the uncertainty range increases by 0.2 Pg C more than the upper bound, reducing the overall uncertainty range. The former lower bound accounted for the additional uncertainty for the bias between surface saturations of δ*p*CO_2_ and *p*CFC‐12 (Tanhua et al., 2009), an estimate that was based on results from the Southern Ocean (Waugh et al., 2006). Here, we explicitly account for the sign and magnitude of these saturation differences in the Arctic.

The remaining uncertainty of the C_ant_ inventory is considered the same as previously estimated by Tanhua et al. (2009, 2008). It includes uncertainties from the Δ/Γ ratio (±5%), from the spatial extrapolation (±5%), and from either very low CFC‐12 concentrations in old waters or supersaturation of CFC‐12 in young waters owing to the decline in atmospheric CFC‐12 since 2002 (±7%). Rajasakaren et al. (2019) also estimated uncertainties from the Δ/Γ ratio and from their lateral extrapolation of $C_{\text{ant}}^{TTD}$ concentrations from the Beringia section to the entire Arctic Ocean. While their uncertainty from the Δ/Γ ratio is also around ±5% (Rajasakaren et al., 2019, figures 8 and 11), they estimate a 50% uncertainty from their spatial extrapolation. Conversely, Tanhua et al. (2009) estimate only a 5% extrapolation uncertainty, an estimate we adopt here. While Rajasakaren et al. (2019) rely on tracer measurements along only the Beringia section in 2005 (23 stations), Tanhua et al. (2009) exploited many more tracer measurements during 1983 to 2005 (535 stations), scaling those to 2005. Although Tanhua et al.'s temporal scaling adds some uncertainty, their use of much more data covering most parts of the four major basins dramatically reduces the overall uncertainty.

## 5 Arctic Ocean Acidification

Using the data‐based $C_{\text{ant}}^{obs}$ concentration estimates from Tanhua et al. (2009), Anderson et al. (2010) calculated that the average depth of the ASH on the Beringia section was 1,890 m in 2005 and that it had shoaled by 190 m between 1765 and 2005. Based on our refined C_ant_ concentration estimates, the distance‐weighted mean ASH along that section is located at 1,950 ± 260 m in 2005 and it has shoaled by 270±60 m during 1765–2005, that is, by 37% more on average than estimated by Anderson et al. (2010). Error bars represent plus or minus one combined standard uncertainty from the propagation of the standard uncertainties of the dissociation constants and the solubility product for aragonite.

**Figure 3:**
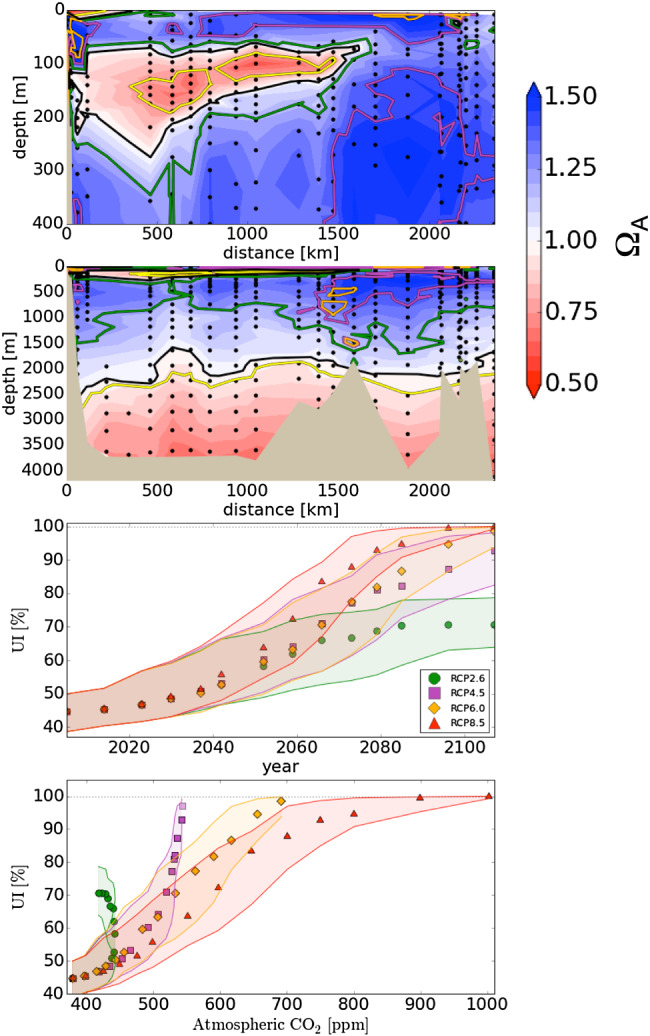
The Ω_A_ and ASH along the Beringia 2005 section over the upper 400 m(top) and over the full water column (upper middle) along with estimates of the time evolution of UI based on four RCP scenarios displayed as a function of year (lower middle) and of atmospheric CO_2_ (bottom). In the top two panels, shading indicates the observed 2005 conditions. Also shown is the ASH for 2005 (black), for the preindustrial back calculation (yellow), and for 2107 for three future scenarios: RCP2.6 (green), RCP4.5 (magenta), and RCP6.0 (orange). Shaded areas in the bottom two panels indicate the combined standard uncertainties propagated from standard uncertainties in the carbonate system dissociation constants and the solubility product for aragonite. The lighter purple marker in the bottom panel is for RCP4.5 in year 2150.

Furthermore, we assessed the spatial variability of the depth of the ASH along the Beringia section. In 2005, that section's deepest ASH (2,450 ± 300m) is found on the continental rise next to the Beaufort Sea, while its shallowest ASH (1,590 ±300 m) is 500 km away in the center of the Canada basin (Figure 3). Shoaling also varies along the section, from as low as 80 m in the southern part of the Canada basin to as much as 550 m in the center of the Canada basin.

For the future scenarios, we applied the same basic approach as used by Anderson et al. (2010), which considers that changes in Ω_A_ are only due to the direct effect of increasing atmospheric CO_2_ on ocean chemistry; effects from climate change are ignored. Thus, we applied the observation‐based TTD obtained for 2005 along the Beringia section to the four future RCP scenarios to estimate the evolution of C_ant_ concentrations and UI along the same section as described in section 2.7 (Figure 3). Between 2005 to 2037, the UI follows the same trajectory under all four RCPs, increasing from 39–50% to 45–64%. Afterwards, it diverges. Under RCP2.6, the UI rises more slowly reaching 63–78% in 2087 and remains constant thereafter. Under the three other RCP scenarios, the UI increases more quickly, for example, in 2100 reaching 80–97% under RCP4.5, 90–100% under RCP6.0, and 97–100% under RCP8.5.

Our idealized TTD‐based estimates were also compared to simulated changes in Ω_A_ from Steinacher et al. (2009), who made projections with the NCAR CSM1.4 climate‐carbon model. During the 21st century, their model is forced under the Special Report on Emissions Scenarios (SRES) B1 and A2, which are similar to the more recent RCP2.6 and RCP8.5 scenarios used here. Steinacher et al. found that in 2100, the entire water column in the Arctic Ocean becomes undersaturated with respect to aragonite under the A2 scenario but not under the B1 scenario. That complete undersaturation under the A2 scenario occurs in 2090 when atmospheric CO_2_ reaches 765 ppm. Likewise, our results suggest that the Arctic water column would become entirely undersaturated by 2100 (Figure 3) under the RCP8.5 scenario, when atmospheric CO_2_ reaches 936 ppm, but not under the lower atmospheric CO_2_ levels of the RCP2.6 scenario, which reaches 443 ppm in 2052 and subsequently declines. Our results also suggest that the Arctic Ocean would eventually become almost entirely undersaturated (UI > 95%) even at atmospheric CO_2_ levels less than 765 ppm.

With the two intermediate RCP scenarios, the UI reaches 93–99% in 2150 under RCP4.5 and 94–100% under RCP6.0. The lowest atmospheric CO_2_ level at which nearly complete undersaturation (UI > 95%) is reached is 540 ppm, the atmospheric CO_2_ value at which RCP4.5 levels out. Under the RCP8.5 scenario, the 540 ppm threshold is reached sooner, in 2051, but the entire Arctic water column does not become undersaturated until 2080 because it takes time to ventilate intermediate and deep waters. These findings emphasize that the year in which the entire Arctic Ocean water column would become undersaturated with respect to aragonite depends not only on reaching a specific atmospheric CO_2_ level but also on the rates of increase and future decline. Complete undersaturation of the Arctic Ocean appears inevitable if atmospheric CO_2_ would reach twice the preindustrial level unless just afterwards there were a sharp decline.

Unlike model studies (Steinacher et al., 2009; Steiner et al., 2013), our future estimates are based on data‐based estimates of the ventilation age and future RCP scenarios of atmospheric CO_2_. This simplified approach suggests that during the 21st century under RCP8.5, the deep ASH would shoal by 900 m, nine times as much as found with the model studies. In contrast, the model studies suggest much greater deepening of undersaturated waters from the surface before there is a merge with the models' more slowly shoaling deep ASH, at which time the entire water column becomes undersaturated. However, these coarse‐resolution ocean models largely underestimate lateral input of C_ant_ into the Arctic Ocean (Terhaar et al., 2019). Their resulting underestimated storage of C_ant_ in intermediate waters implies that they also underestimate the shoaling of the deep ASH.

Our TTD‐based estimates of increasing C_ant_ concentrations and resulting acidification during the 21st century account for future invasion of C_ant_ but neglect climate change and its affect on the ocean. Yet the Arctic Ocean is already experiencing changes in sea‐ice extent (Serreze & Stroeve, 2015) and primary production (Arrigo & van Dijken, 2015). During this century, the Arctic Ocean is also projected to experience large increases in summertime sea‐surface temperature (Carton et al., 2015). All of these changes will affect Arctic Ocean uptake of C_ant_ and hence its acidification. Steinacher et al. (2009) estimate with their model projection that the dominant climate‐change effect on Ω_A_ is a reduction due to dilution of *C*_T_ and *A*_T_ from sea‐ice melt. Climate change is also expected to alter the Arctic Ocean circulation and the associated transport of C_ant_ into the deep Arctic Ocean, although the primary driver of overall change in Ω_A_ is the increase in C_ant_. Lique et al. (2018) found that the climate‐change‐induced brine rejection from sea‐ice melt intensified deep‐water convection in the Eurasian basin in the HiGEM climate model forced under a 4×CO_2_ scenario. Some indication for this projected increase in deep‐water formation in the Arctic Ocean appears to have already been observed, namely, through an increased contribution of Arctic waters to deep water masses in the Nordic Seas (Langehaug & Falck, 2012; Somavilla et al., 2013). More Arctic deep convection would further enhance Arctic storage of C_ant_ and thus deep‐water acidification and shoaling of the deep ASH. Steinacher et al. (2009) found that the effects of climate change will enhance the reduction of Ω_A_ in the Arctic Ocean. Thus, our estimate of the future reduction in Arctic Ocean Ω_A_, which neglects climate change, should be considered as a lower bound. Despite the uncertainties associated with the idealized nature of our study, including its neglect of climate change and observational uncertainties, it is consistent with previous studies in estimating that in coming years the Arctic Ocean will experience widespread undersaturation with respect to aragonite, a dramatic chemical change that is expected to degrade the Arctic Ocean ecosystem (Fabry et al., 2008; Gattuso & Hansson, 2011; Riebesell et al., 2013).

## 6 Conclusion

Our model‐based evaluation has demonstrated that the TTD approach underestimates C_ant_ concentrations in the Arctic Ocean by up to 12% depending on the water mass considered. These underestimates occur throughout the water column, being tied through water‐mass formation to biases in surface waters. The surface C_ant_ concentrations estimated by TTD are generally too low because of that approach's assumption of perfect gas equilibrium between the surface ocean and the atmosphere for both δ*p*CO_2_ and *p*CFC‐12. In contrast, our ocean model produces surface‐ocean δ*p*CO_2_ that is supersaturated with respect to atmospheric δ*p*CO_2_ at the time of deep‐water formation, while also producing surface ocean *p*CFC‐12 that is undersaturated relative to atmospheric *p*CFC‐12. Not accounting for these contrasting tendencies biases the TTD approach. After adjusting the 2005 Arctic Ocean C_ant_ inventory from Tanhua et al. (2009) for these biases, the best estimate of that inventory increases from 3.0 to 3.3 Pg C while the uncertainty range moves from 2.5–3.3 to 3.0–3.6 Pg C.

By using the bias‐corrected TTD approach with future atmospheric CO_2_ scenarios, we provide an estimate of future conditions for an idealized case where effects from climate change are neglected. Results from that simplified analysis suggest that the Arctic Ocean would eventually become entirely undersaturated with respect to aragonite unless atmospheric CO_2_ were stabilized below 540 ppm. Avoiding an entirely undersaturated Arctic Ocean would thus not be possible under the RCP4.5 scenario, which maintains that CO_2_ level after 2100. Effects from climate change would probably reduce that atmospheric CO_2_ threshold. Retaining some Arctic Ocean waters that are not corrosive to aragonite appears feasible only under more optimistic scenarios such as RCP2.6. To better characterize the atmospheric CO_2_ stabilization threshold at which the Arctic Ocean would eventually become entirely undersaturated with respect to aragonite, more studies are needed with Earth System Models forced under stabilization scenarios beyond 2100.

## Data Availability Statement

Model output was analyzed on CICLAD at IPSL and is openly accessible on the ODATIS‐supported center SEANOE (https://www.seanoe.org/data/00628/74022/).

## Supporting information

Supporting Information S1
