# Supplementary material for: Evaluation of Data‐Based Estimates of Anthropogenic Carbon in the Arctic Ocean
Source: J Geophys Res Oceans. 2020 Jun 7;125(6):e2020JC016124. doi: 10.1029/2020JC016124 (PMC7380301; doi:10.1029/2020JC016124)
Supplement: Supplementary file 1 — Supporting Information S1 [file JGRC-125-e2020JC016124-s001.pdf]

Supporting Information for "Evaluation of data-based estimates of anthropogenic carbon in the Arctic Ocean"

J. Terhaar[1,2], T. Tanhua[3], T. Stöven[3], J.C. Orr[1], L. Bopp[4]

[1]Laboratoire des Sciences du Climat et de l'Environnement, LSCE/IPSL, CEA-CNRS-UVSQ, Université Paris-Saclay, 91191 Gif-sur-Yvette, France

[2]Biogeochemistry and Earth System Modelling, Department of Geoscience, Environment and Society, Université Libre de Bruxelles, Belgium

[3]Helmholtz Centre for Ocean Research Kiel, GEOMAR, Kiel, Germany

[4]LMD/IPSL, Ecole Normale Supérieure / PSL Research University, CNRS, Ecole Polytechnique, Sorbonne Université, Paris, France

Contents

1. Table S1
2. Figures S1 to S2

Table S1. Water mass analysis and corresponding corrective factors

	SPW	WPW[a]	AW	BSW	SW
T (°C)	>-1	<-1	>0	<0	
S	31.0–33.0	32.4–33.0	>34.0	>34.6	
Depth (m)	50–100	50–500	>50	>50	0–300
Corrective factor (%)	7±2	12±3	4±2	12±2	
Number of data points	54	228	3175	3127	3139

[a] WPW is geographically limited to regions south of 85°N and east of 90°E and west of 90°W.

Corresponding author: J. Terhaar, jens.terhaar@lsce.ipsl.fr

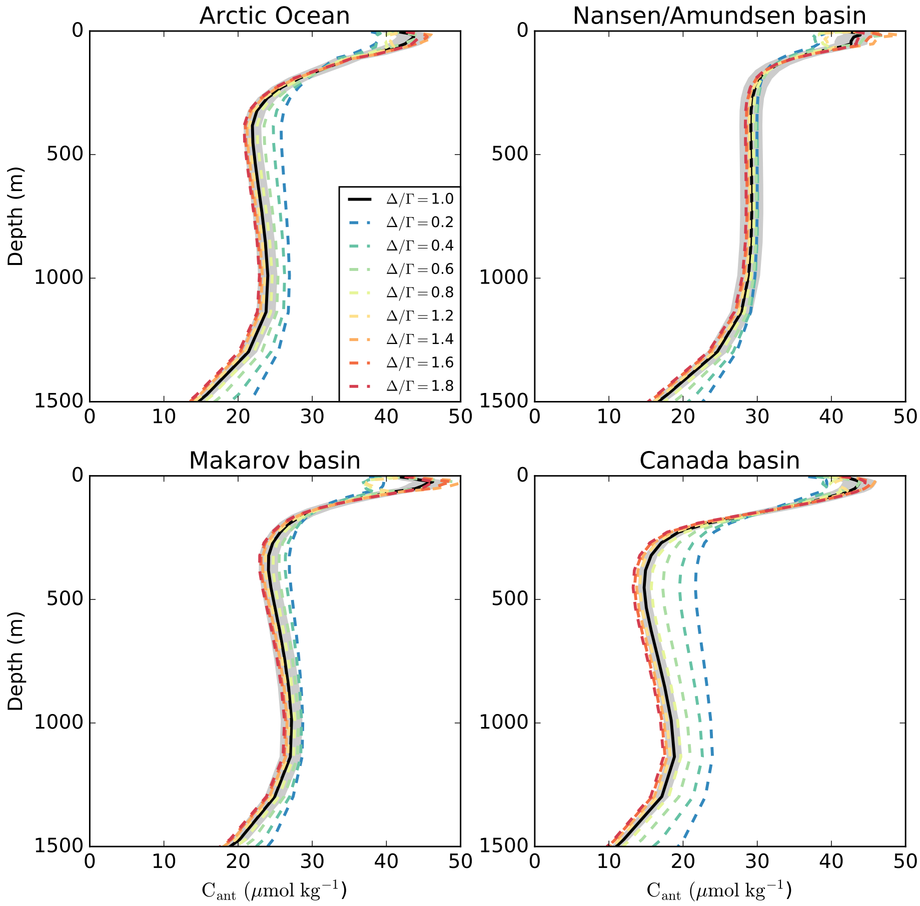

Figure S1. Vertical profiles of C_{ant}^{TTD} over the top 1500 m computed from CFC-12 simulated in ORCA025 using different Δ/Γ ratios, including the classic ratio of 1.0 (black solid) and other values between 0.2 to 1.8 (colored dashed). Results are shown as averages for the entire Arctic Ocean as well as the the Nansen and Amundsen basin, the Makarov basin, and the Canada basin. The $\pm5\%$ uncertainty range is shown in grey.

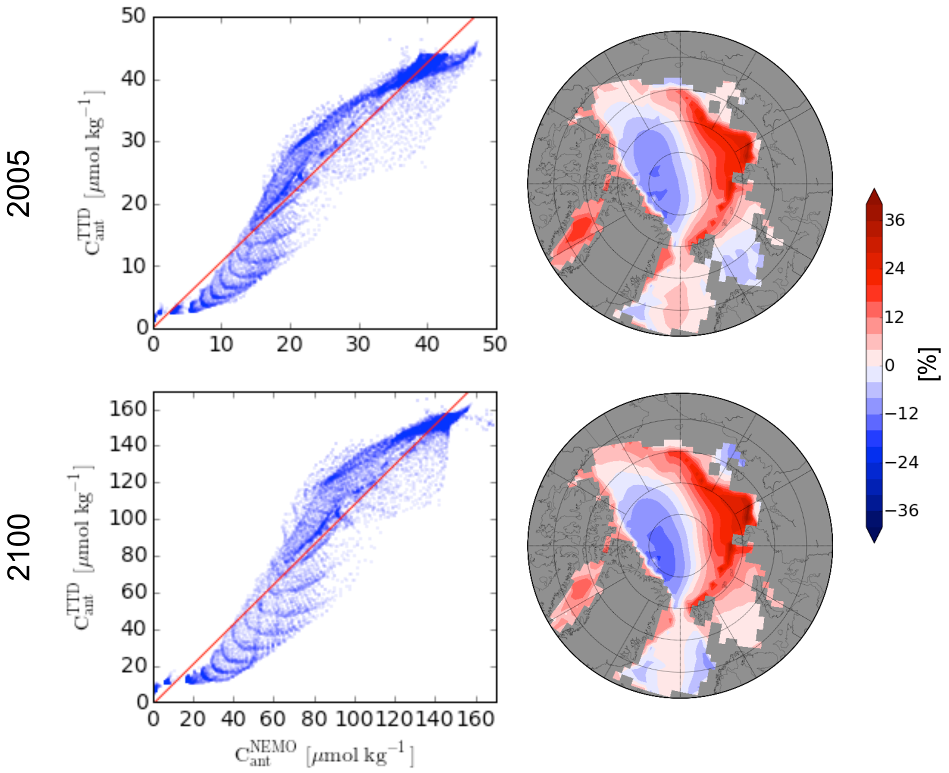

Figure S2. Point-by-point comparison between C_{ant}^{TTD} and C_{ant}^{NEMO} calculated from CFC-12 in ORCA2 for 2005 (top left) and 2100 (bottom left) with the respective linear fit (red line) along with maps of relative differences [%] between vertical integrated C_{ant}^{TTD} and C_{ant}^{NEMO} in 2005 (top right) and 2100 (bottom right).
